# Synthetic neutrophil extracellular traps dissect bactericidal contribution of NETs under regulation of α-1-antitrypsin

**DOI:** 10.1126/sciadv.adf2445

**Published:** 2023-04-28

**Authors:** Ting Yang, Jinlong Yu, Tasdiq Ahmed, Katherine Nguyen, Fang Nie, Rui Zan, Zhiwei Li, Pei Han, Hao Shen, Xiaonong Zhang, Shuichi Takayama, Yang Song

**Affiliations:** ^1^ School of Material Science and Engineering, Shanghai Jiao Tong University, Shanghai 200240, China.; ^2^ Wallace H Coulter Department of Biomedical Engineering, Georgia Institute of Technology and Emory School of Medicine, Atlanta, GA 30332, USA.; ^3^ Department of Orthopedics, Shanghai Sixth People’s Hospital Affiliated to Shanghai Jiao Tong University School of Medicine, Shanghai 200233, China.; ^4^ Renji Hospital affiliated to Shanghai Jiao Tong University, Shanghai 200127, China.; ^5^ Shanghai Engineering Research Center of Biliary Tract Minimal Invasive Surgery and Materials, Shanghai 200032, China.

## Abstract

Deciphering the complex interplay of neutrophil extracellular traps (NETs) with the surrounding environment is a challenge with notable clinical implications. To bridge the gap in knowledge, we report our findings on the antibacterial activity against *Pseudomonas aeruginosa* of synthetic NET-mimetic materials composed of nanofibrillated DNA-protein complexes. Our synthetic system makes component-by-component bottom-up analysis of NET protein effects possible. When the antimicrobial enzyme neutrophil elastase (NE) is incorporated into the bactericidal DNA-histone complexes, the resulting synthetic NET-like structure exhibits an unexpected reduction in antimicrobial activity. This critical immune function is rescued upon treatment with alpha-1-antitrypsin (AAT), a physiological tissue-protective protease inhibitor. This suggests a direct causal link between AAT inhibition of NE and preservation of histone-mediated antimicrobial activity. These results help better understand the complex and, at times, contradictory observations of in vivo antimicrobial effects of NETs and AAT by excluding neutrophil, cytokine, and chemoattractant contributions.

## Teaser

Minimalist NETs are synthesized as biomaterials to decipher their in-tissue antimicrobial activity against P. aeruginosa.

## INTRODUCTION

As part of our innate immune response, the release of neutrophil extracellular traps (NETs), composed of DNA conjugated with a variety of bactericidal proteins and enzymes, works to disarm invading pathogens (*1*–*3*). NETs were first reported in 2004 as a host defense mediator designated for bacteria trapping and killing; the antimicrobial activity of NETs has since been extensively investigated against a variety of bacteria, fungi, viruses, and parasites (*1*, *4*, *5*). In hopes of identifying the critical bactericidal protein(s) within NETs, researchers have used antibodies or enzyme inhibitors to target select proteins and strategically determine their individual contributions to bacteria killing (*6*). However, these bioanalytical methods are costly, laborious, and semiquantitative, and their top-down approach itself may produce artifacts stemming from the loss of compository elements essential to NET integrity (*7*). Many of these NET proteins are bactericidal at minimum inhibitory concentrations (MICs) for specific bacteria but are present at sub-MIC levels in NETs (*8*). In addition, NETs are continuously degraded by enzymes and phagocytes in the physiological environment, making the quantification of NET proteins and elucidation of their contributions a challenging task (*9*). Although specific NET proteins have been isolated for study, their antimicrobial activity may deviate from that when incorporated in NETs. For instance, while many antimicrobial proteins kill bacteria via their ionized amino groups, these cationic groups experience a screening effect in the presence of DNA, dampening their activity in NETs (*10*). Therefore, traditional analytical methods are insufficient for understanding the structure, composition, and dose-dependent antimicrobial activity of NETs.

The emergence of nature-inspired synthetic biomaterials can enable systematic analysis of NET antimicrobial activity by tailoring NET structure and composition. Stephan *et al.* (*11*) proposed that the antimicrobial performance of NETs could be simulated using DNA-protein complexes. They prepared a DNA-LL37 complex and identified their potency in killing phagocytosed bacteria in macrophages. A key question about NET mimetics is whether their DNA nanofiber meshwork can be built such that they trap bacteria as efficiently as NETs. We have previously reported a NET-mimetic material synthesis approach by complexing DNA with histones, forming web-like microstructures (abbreviated as “microwebs”) (*12*). Similar to NETs, microwebs exhibit excellent bacterial trapping capacity, dose-dependent antimicrobial activity, and immune-activating functionality (*13*). However, NETs have far more complicated proteomics and antimicrobial behaviors than microwebs, not only because of the collaborative or antagonistic mechanisms of action among different NET proteins but also because of their variable antimicrobial activity in different tissue environments.

To better reproduce the antimicrobial activity of NETs, we present our synthesis of next-generation NET-mimetic biomaterials with a biochemical formulation representing a broader range of NET constituents. Important antimicrobial enzymes, such as neutrophil elastase (NE) and myeloperoxidase (MPO) are incorporated into DNA-histone complexes (DHCs) to evaluate their contributions to bacteria killing in NET-like structures. This bottom-up biomimetic material synthesis approach provides a defined model to link the antimicrobial activity of NETs with their structure and composition. In addition, we investigate the tissue-derived enzyme inhibitor alpha-1-antitrypsin (AAT) in regulating the antimicrobial activity of NETs and, in a lung epithelial context, to better understand the interplay between NETs and tissue environment.

## RESULTS

**Fig. 1.**
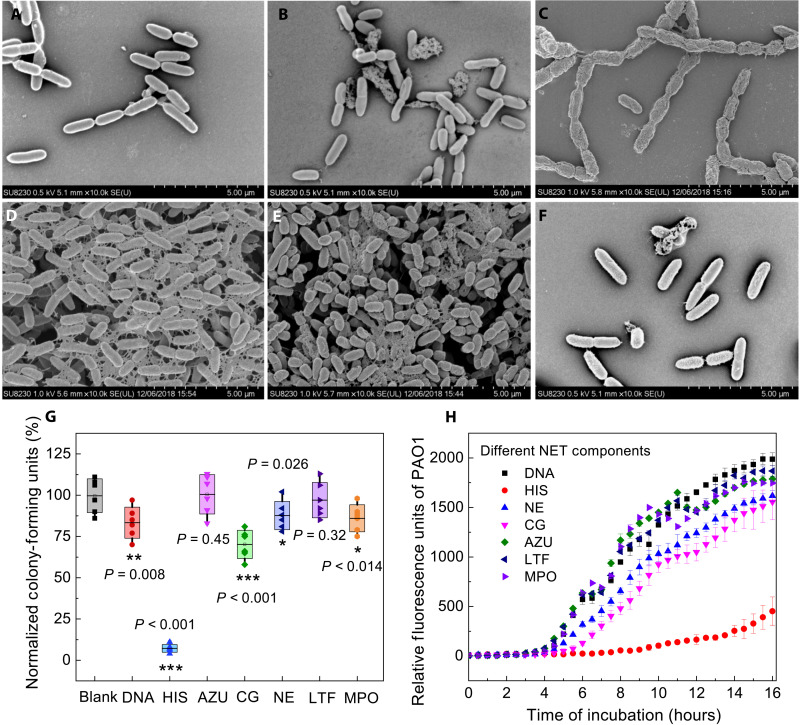
Antimicrobial activity of individual NET-derived antimicrobial proteins or DNA against *P. aeruginosa* PAO1. Scanning electron microscopy (SEM) images of PAO1 cultured in SCFM with (**A**) no protein or DNA, (**B**) DNA (600 μg ml^−1^), (**C**) histone (553 μg ml^−1^), (**D**) CG (35 μg ml^−1^), (**E**) NE (80 μg ml^−1^), or (**F**) MPO (42.8 μg ml^−1^). (**G**) Numerated colony-forming units (CFUs) of PAO1 after coculture with different NET components for 2 hours. Analysis of variance (ANOVA) followed by Tukey’s test was used for statistical analysis: ns, not significant; *P < 0.05, **P < 0.01, and ***P < 0.001; *N* = 6 for each condition. (**H**) Growth curves of PAO1 in SCFM supplemented with a single NET component. HIS, histone.

To acquire a minimalist biomaterial model of NETs, we first screened different antimicrobial proteins found in NETs against *Pseudomonas aeruginosa*, a respiratory pathogen causing lung infection in cystic fibrosis (CF), chronic obstructive pulmonary disease (COPD), and AAT deficiency (AATD) diseases (*14*–*17*). A representative bacteria strain PAO1 was cultured in synthetic CF medium (SCFM) to recapitulate the growth rates and gene expression profiles observed for bacteria grown in neutrophil-enriched diseased lungs (*18*). To evaluate the antimicrobial activity of NET-like compounds, SCFM was supplemented with each of the major NET components, as shown in Fig. 1 (A to F). Salmon DNA, with its length close to the DNA fragments found in CF sputum, was supplemented into SCFM (at 0.6 mg ml^−1^), at levels equivalent to the average extracellular DNA (eDNA) level in CF sputum (*19*). The physiological concentrations of NET proteins, including histone, azurocidin (AZU; also known as cationic protein 37), NE, MPO, cathepsin G (CG), and lactoferrin (LTF) were estimated using their stoichiometric ratios to DNA in NETs, as previously reported from mass spectrometry analysis of neutrophil-produced NETs (see Table 1) (*7*).

**Table 1.** Compositions of DHCs used to simulate NETs. The concentration of DNA is determined from the average DNA concentration in the sputum of patients with CF (*20*). The histone fraction is calculated from a combination of H_2_A, H_2_B, H_3_, and H_4_ (*21*).

Component	DNA	HIS	LTF	NE	MPO	AZU	CG
Concentration (μg ml^−1^)	600	553	111	80	42.8	33.8	35.3
Weight ratio to DNA (%)	100	92.3	18.5	13.3	7.1	5.6	5.9

After PAO1 (10^6^ ml^−1^) was cultured in SCFM supplemented with individual NET-derived components for 2 hours, we examined bacterial cell wall intactness using scanning electron microscopy (SEM). The number of bacterial colony-forming units (CFU) was also numerated to quantitatively evaluate the antimicrobial potency of each NET component. Free histone, NE, CG, MPO, and eDNA were identified as bactericidal components against PAO1 (*22*). Histone (500 to 600 μg ml^−1^) was observed to disrupt the bacterial cell wall and distort PAO1 into filamentous shapes (Fig. 1C). Numeration of the histone-treated bacterial culture reveals that more than 90% of PAO1 were killed, suggesting a strong bactericidal capability. CG also induces notable bacterial lysis within the physiological range of 30 to 50 μg ml^−1^ (*23*). In contrast, other NET components, including DNA, NE, and MPO, are less potent against PAO1. eDNA was observed to induce small levels of bacterial lysis only when its concentration exceeds 400 μg ml^−1^ (*24*). The enzyme MPO (42 μg ml^−1^), which catalyzes the conversion of chloride ions and hydrogen peroxide (H_2_O_2_) into bleach within neutrophil phagosomes (*25*), only kills less than 20% of bacteria in the extracellular environment (Fig. 1, G and H). AZU is a cationic chemoattractant of monocytes, and LTF is an antibiofilm component failed to kill PAO1 at physiological concentrations (*26*, *27*). Overall, histone and CG are viewed as the most potent antimicrobials among the tested NET proteins at physiological concentrations within NETs.

**Fig. 2.**
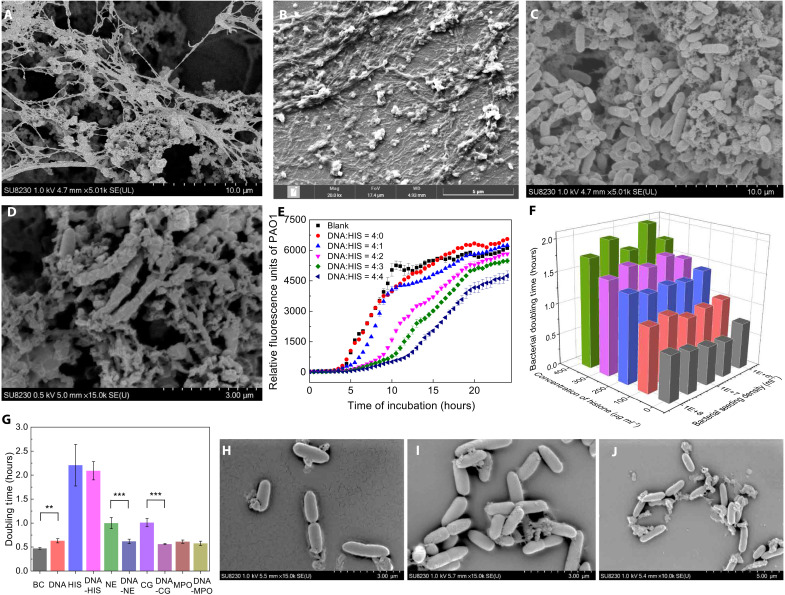
Antimicrobial activity of synthetic DNA-protein complexes against PAO1. (**A**) SEM images of DHCs and (**B**) NETs. (**C**) PAO1 entrapped in DHC after coculture for 1 hour. (**D**) The entrapped bacteria are lysed by the DHC after exposure for 2 hours. (**E**) Growth curves of PAO1 in SCFM supplemented with DHCs with varying weight ratios. (**F**) Bacterial doubling time increases with histone concentration in the DHCs, independent of bacterial seeding density. An increase in doubling time indicates antimicrobial action. (**G**) Bacterial doubling time in SCFM supplemented with different combinations of DNA-protein complexes. ANOVA followed by Tukey’s test was used for statistical analysis: **P < 0.01 and ***P < 0.001; *N* = 6 for each condition. SEM micrographs of PAO1 cocultured with (**H**) DNA-CG, (**I**) DNA-NE, and (**J**) DNA-MPO complexes in SCFM for 2 hours. Scale bars, (A) 10 μm, (B), (C), and (J) 5 μm, and (D), (H), and (I) 3 μm.

We also separately mixed DNA with each bactericidal NET protein at stoichiometric ratios similar to NETs, producing a set of DNA-protein complexes. The DHCs have a web-like architecture (Fig. 2A) resembling NETs (Fig. 2B) at both the microscale and nanoscale, where numerous DNA nanofibers assemble into web-like fibers with granular structures cross-linking them. The thickness of DNA fibrils in the DHC can be tuned from 200 nm to less than 50 nm by reducing the DHC concentration from 0.6 to 0.1 mg ml^−1^ (fig. S1). After incubation with DHCs for 1 hour, most *P. aeruginosa* were entrapped in the DHCs (Fig. 2C). Subsequent culture of these ensnared bacteria (*t* = 2 hours) leads to substantial cell lysis (Fig. 2D). Bacterial growth curves were generated by culturing seeded PAO1 (10^7^ CFU ml^−1^) in SCFM with DHCs for 24 hours (Fig. 2E). The weight ratio ω of histone to DNA in the DHCs was varied to simulate the fluctuating levels of histone within NETs because of degradation in different tissue environments (*28*). To determine the bacterial generation time, the bacterial relative fluorescence units (RFUs) as a function of time are drawn on a logarithmic scale, log (RFU of PAO1) ~ *t*. The bacterial doubling time is estimated from the ratio of log_2_ to the slope of the growth curve in the exponential stage. As deduced from the growth curves, the bacterial doubling times (*T*g) are delayed threefold when the ω(histone):ω(DNA) ratio is increased from 0:1 to 1:1 (Fig. 2F), independent of bacterial seeding density ranging from 10^6^ to 10^8^ CFU ml^−1^. When the DHCs were degraded by adding a sufficient amount of deoxyribonuclease I (DNase I) before bacteria seeding, the bacteria proliferation rate was comparable to that in the blank control group (see fig. S2). While *Pseudomonas* secrete endonucleases to evade NET-mediated killing (*29*), PAO1, however, does not generate enough endonuclease to degrade DHCs or influence their bactericidal activity under the current synthetic settings. Our observations suggest that bactericidal histone conjugates with DNA meshwork to inhibit bacterial proliferation. We also find that other antimicrobial proteins such as NE, CG, and MPO almost completely lose their bactericidal activity after complexing with DNA, as shown by the growth curves in fig. S3 and doubling times in Fig. 2G. SEM observations reveal that the obtained DNA-NE, DNA-MPO, and DNA-CG complexes are amorphous nanoparticles. These nanoparticles adhere to the bacterial cell wall, but no distinct cell wall damage could be identified (Fig. 2, H to J). The above findings suggest that DHCs, rather than other DNA-protein combinations, essentially recapitulate the structural features and antimicrobial function of NETs against PAO1.

**Fig. 3.**
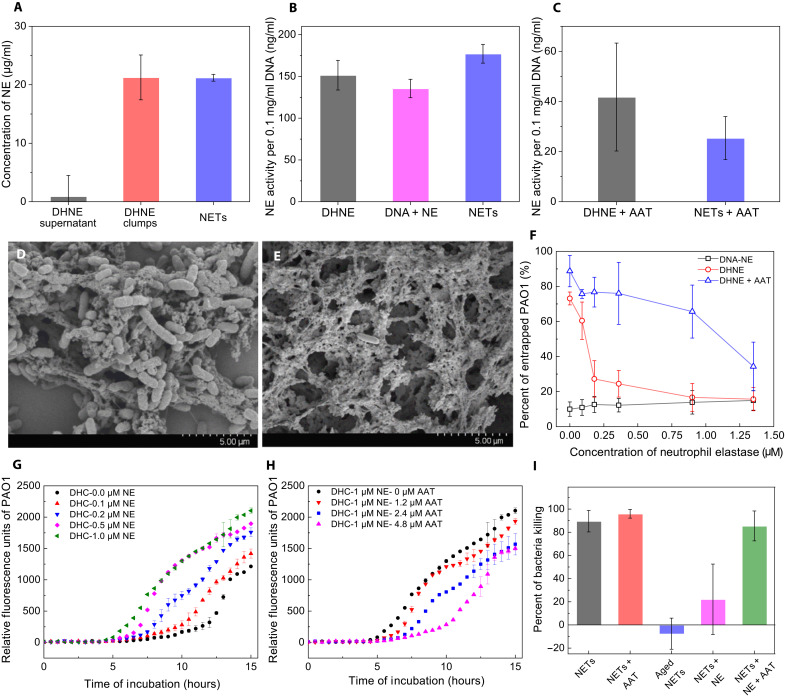
High NE activity impairs the bacteria-trapping and bactericidal activity of DHNEs. (**A**) ELISA results showing the amount of NE present in synthetic DNA-histone (DNA-HIS) complexes and in natural NETs. (**B**) A test on the enzyme activity of NE in DNA-NE complexes, DHNEs, and NETs. (**C**) AAT (1 μM) deactivates NE in DHNEs and NETs. (**D** and **E**) SEM images showing the bacterial trapping ability of DHNE (D) with 5 μM AAT and (E) without NE inhibitor. Scale bars, 5 μm. (**F**) The relative numbers of entrapped bacteria, in response to NE, were quantified from bacteria fluorescence intensity. Growth curves of PAO1 in SCFM supplemented with (**G**) DHNEs and (**H**) DHNEs supplemented with AAT. (**I**) The bactericidal activity of NETs changes with NE activity in SCFM. High NE activity leads to impaired antimicrobial potency, as shown by numerated CFUs of PAO1 after coculture with NETs in SCFM supplemented with extra NE or AAT. A substantial loss of bactericidal activity was observed when NETs are either aged for 48 hours or given an additional 1 μM NE. Inhibition of NE activity by adding AAT (1 μM) rescued the bactericidal activity of NETs against PAO1.

We further asked whether different protein combinations in the DNA-protein meshes operate synergistically or antagonistically to kill *P. aeruginosa*. MPO and NE, two key NET proteins with different antimicrobial mechanisms, were separately mixed with DHCs to create more complicated NET-like structures, termed DNA-histone-NE complexes (DHNEs) and DNA-histone-MPO complexes (DHMPOs) (*1*, *2*, *30*). Incorporation of these enzymes into the DNA meshes were verified using the relevant enzyme-linked immunosorbent assay (ELISA), and their enzymatic activity was assessed using commercial assay kits (Fig. 3A and fig. S4A). Both NE and MPO can complex with eDNA through electrostatic interactions, resulting in decreased enzyme activities. When keeping DNA amounts consistent, the synthesized DHNEs exhibit comparable NE enzymatic activity as in NETs collected from healthy donor neutrophils (Fig. 3B). To model the inhibition of serine proteases in lung lining fluids, we also included the most prevalent NE inhibitor, AAT (α_1_-AT) (*31*–*34*). The presence of 1 μM AAT inhibited the NE activity by 58 ± 21% in DHNEs [both groups contain DNA (0.2 mg ml^−1^)] and by 74 ± 8% in NETs (Fig. 3C). A further increase in AAT (up to 5 μM) almost neutralizes the enzymatic activity of NE (>90%).

The effect of NE activity on the overall DHNE antimicrobial activity was then evaluated by seeding PAO1 over DHNEs and measuring their bacterial trapping efficacy. While NE is believed to mediate the innate host response against *P. aeruginosa*, unexpectedly, DHNEs trap a lower density of PAO1 compared to the NE-free DHCs, as shown by SEM in Figs. 3D and 2B. By labeling PAO1 with the red fluorescent protein mCherry, the proportion of entrapped bacteria can be quantified from the fluorescence in the DHNEs relative to the whole culture medium. The bacterial trapping efficacy of DHNEs decline with elevating NE activity (Fig. 3D). Consequently, free bacteria remain alive and the overall bacterial proliferation rate recovers (Fig. 3, F and G). Notably, when the concentration of NE is raised to high levels equal to that observed in CF lung lining fluids (≥1 μM), the resulting DHNEs completely lose their PAO1 killing potency. If the enzymatic activity of NE is blocked by AAT before mixing with DHCs, then the bacterial trapping and killing potency of the DHNEs can be preserved to a large extent (Fig. 3, F and H). Note that AAT alone does not change the proliferation rate of PAO1 (fig. S5). The preservation of antimicrobial potency after addition of AAT is attributed to reduced elastase activity, which is consistent with previous reports (*35*). This NE enzyme activity-dependent bactericidal activity was also confirmed using neutrophil-produced NETs. When NETs were immersed in SCFM supplemented with 1 μM NE, the bactericidal activity of NETs dropped sharply. In contrast, the bactericidal activity was commendably preserved if suspended in a mixture of 5 μM AAT and 1 μM NE (Fig. 3I). The NET bactericidal activity was also diminished when NETs were incubated in AAT-free solution for 48 hours (aging of NETs; see Fig. 3I). These observations led us to conclude that the bactericidal activity of NETs can be regulated by the enzyme activity of NE, which is determined by the balance between NET-derived NE and its inhibitors in the tissue environment. Other NET-derived serine proteases, such as CG, could also induce a pronounced decline in bactericidal activity; however, the concentrations of these proteases in NETs are comparatively low. Incorporation of MPO, an oxidase necessary for NET formation, did not change the bactericidal activity of DHCs, even in the presence of 5 μM H_2_O_2_ to facilitate bleach production (fig. S4B).

**Fig. 4.**
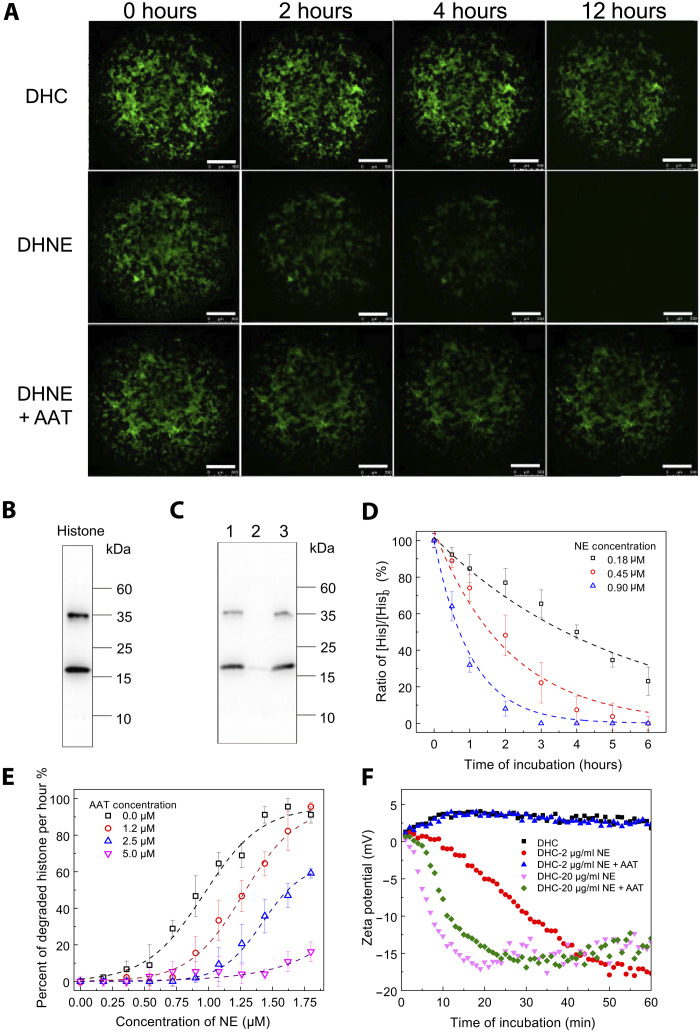
NE degrades histone in the DHNEs. (**A**) Fluorescence micrographs showing NE-mediated degradation of histone. Scale bars, 500 μm. (**B**) Western blot of histone using H3 monoclonal antibody. (**C**) NE degrades histone in DHCs. Lane 1: DHNE + AAT; lane 2: DHNE; lane 3: DHC. (**D**) Degradation kinetics of DHCs driven by the NE concentration. (**E**) AAT inhibits NE-mediated degradation of DHCs. (**F**) Measures of DHC zeta potentials when supplemented with different concentrations of NE and AAT.

Because histone is the most potent antimicrobial protein, the neutralizing effects of NE prompted us to speculate that NE may alter NET proteomics by degrading histone, leading to impaired antimicrobial activity (*28*, *36*). To test this hypothesis, histone was labeled with fluorescein isothiocyanate (FITC) fluorophore and used to form complexes that were then dehydrated. After rehydrating FITC-DHCs with 0.1 μM NE, we observed the breakdown of DHCs, as fluorescence faded over time in a light-controlled environment (Fig. 4A). In contrast, both the NE-free DHCs and the AAT-containing DHNEs retain fluorescence. The NE-mediated degradation of histone in DHCs was also confirmed by labeling histone H3 with monoclonal antibody and running Western blots (Fig. 4, B and C). Our findings are in agreement with previous observations (*22*, *37*). To better quantify the NE-mediated degradation of histone, we first homogenized non-fluorescent DHCs into a colloidal suspension by ultrasonication, followed by an immediate measurement of its turbidity with a UV-Vis spectrophotometer. The optical density (OD_600_) of the DHNE mixture decreases over time, as a result of NE-mediated degradation of histone (Fig. 4D). The OD_600_ of homogenized DHNEs linearly decreases with histone concentration, as shown in fig. S6. Eventually, the mixture becomes transparent with an OD value close to zero. This dynamism in homogenized DHC suspension turbidity allows us to evaluate the NE-mediated degradation kinetics of histone. The degradation rate of histone in the DHNEs increased with the concentrations of NE and histone (Fig. 4D and fig. S7). A supplement of NE inhibitor (1 to 5 μM) delays histone degradation in DHNEs (Fig. 4E) and neutrophil-produced NETs (fig. S8). Note that it is inappropriate to directly predict the degradation cycle of histone in NETs by using our DHNEs model, as NET-derived NE (and other serine proteases) degrades a variety of other NET proteins, as well as extracellular matrix (ECM). However, DHNEs provide a simplified model to reflect the degradation of bactericidal components in NETs, which can be modulated by the activity of serine proteases. The NE-mediated degradation of histone explains the declined bacterial trapping efficacy of DHNEs as the NE concentration increases. As histone utilizes its polycationic surface to entrap bacteria, the incorporation of 0.1 μM NE could decrease the zeta potential of the DHNEs from +4 mV to −15 mV (Fig. 4F). Thus, the electrostatic repulsion between DHNEs and the bacterial cell walls is increased, so as to prevent bacterial trapping. If sufficient AAT is mixed with NE before DHNE formation, then the DHNEs maintain positive zeta potentials (Fig. 4F), leading to the well-preserved bacterial trapping and killing potency. The above results suggest that AAT regulates the antimicrobial potency of DHNEs through inhibition of NE-mediated degradation of histone.

**Fig. 5.**
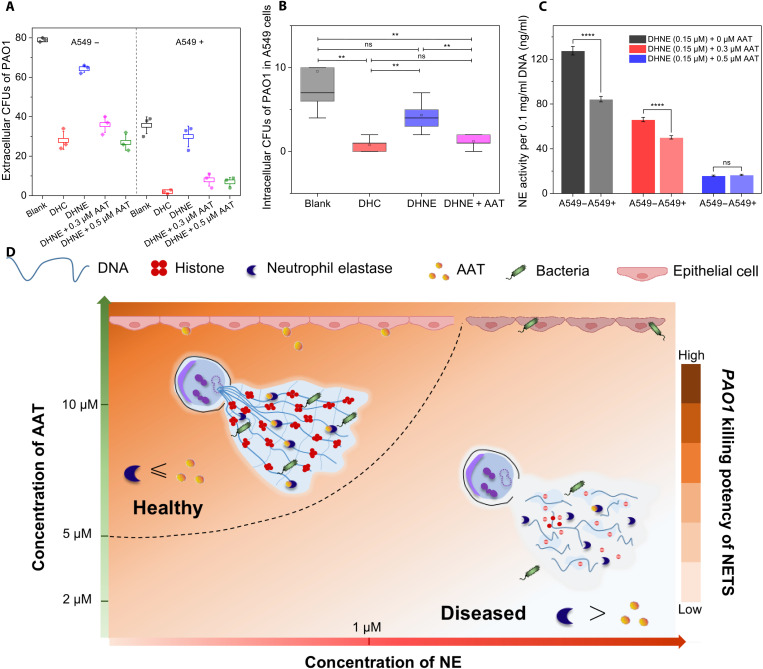
Antimicrobial collaboration between A549 lung epithelial cells and synthetic NETs is regulated by NE and AAT levels. (**A**) Antimicrobial activity of DHNEs against PAO1 in presence and absence of A549 cells. (**B**) CFU counts of PAO1 inside A549 cells after coculture with either DHCs, DHNEs, or DHNEs with AAT. ANOVA followed by Tukey’s test was used for statistical analysis: **P < 0.01; *N* = 9 for each condition. (**C**) A549 cells suppress the enzymatic activity of NE in DHNE. Concentration of NE is 0.15 μM; ANOVA followed by Tukey’s test was used for statistical analysis: ****P < 0.0001; *N* = 3 for each condition. (**D**) Schematic showing that an imbalance between NE and its inhibitor AAT can impair the antimicrobial collaboration between NETs and epithelial cells.

To better simulate the antimicrobial collaboration between NETs and tissue environment, the antimicrobial behavior of DHNEs was further evaluated by coculturing PAO1 with A549 human lung epithelial cells, followed by measuring PAO1 CFU counts inside and outside of A549 cells. Extracellular CFU counts declined in the presence of A549 cells, and the observed antimicrobial activity could be strengthened with DHNEs, but largely upon exogenous supplementation of AAT (Fig. 5A). Compared to DHCs, CFU counts of PAO1 inside A549 cells was higher for DHNEs, and the addition of exogenous AAT supplementation restored DHNE antimicrobial activity to similar levels of DHCs alone (Fig. 5B). These observations suggest that AAT protects the antimicrobial activity of DHNEs. Note that A549 cells secrete AAT and potentially other factors to suppress NE activity (Fig. 5C) (*38*). A549 cells alone, however, fail to fully inhibit NE activity. This is consistent with physiology in that AAT from the liver and other sources must combine with lung epithelium–produced AAT to sufficiently inhibit NE activity. In addition to antibacterial effects, AAT supplementation reduces DHNE cytotoxicity toward epithelial and endothelial cells (fig. S9). Overall, these results show support for the collaborative roles of epithelial cells, other tissue sources of AAT, and DHNEs in eliminating bacteria. In healthy lung tissue, NE and its inhibitor AAT are proposed to provide a balanced homeostatic level of bactericidal proteins in NETs. Under conditions of hyperactive NE, however, the balance is broken, causing injuries to epithelial/endothelial barriers and impairing the antimicrobial activity of NETs (Fig. 5D).

## DISCUSSION

*P. aeruginosa* is a pathogen that induces NETosis and is susceptible to NET-mediated capture and killing (*39*). Histones, the most abundant proteins in NETs, exhibit bactericidal activity against *P. aeruginosa* (*40*). In agreement with prior reports, we confirmed that histones are the most potent antimicrobials against PAO1 among a list of major NET proteins. In addition, the DNA-histone meshwork is an antimicrobial structure, although DNA itself is not a strong antimicrobial. Halverson *et al.* (*24*) have stated that DNase treatment can dissolve DNA structures and prevent NET-mediated bacterial capture and killing of *Staphylococcus aureus* and *P. aeruginosa*. Similarly, we find that DNase treatment can break down the DHC structure, and the antimicrobial activity of DHCs is completely lost after DNase treatment (*5*). DNase I cleaves the phosphodiester linkages of DNA and may underpin the charge neutralization of the phosphate-terminated polynucleotides with histone, which further blocks the histone bactericidal activity (*41*, *42*). Other DNA binding proteins show similar surface charge–dependent antimicrobial activity. For example, while isolated MPO, NE, and CG are bactericidal components, the DNA-MPO, DNA-NE, and DNA-CG complexes fail to puncture the bacterial cell wall or affect their viability. Instead, these DNA-protein complexes may fight against PAO1 using mild antimicrobial strategies. For instance, NE represses the expression of flagellin in *P. aeruginosa* caught in NETs (*43*) and restrict their swimming motility without killing them (*44*). MPO facilitates the intracellular generation of hypochlorous acid and efficiently kills *P. aeruginosa* inside phagosomes (*45*), yet its antimicrobial potency appears to be restricted by its complexation with DNA in extracellular spaces. Although DHCs are less effective in directly killing PAO1 than free histone, they are better phagocytosed by dendritic cells, leading to increased immune response activation (*13*). NET structures may also help suppress bacterial toxin spreading by entrapment followed by immune cell uptake, whereas antimicrobial peptides often lyse bacteria, causing a release of inflammatory toxins into the surrounding environment (*1*). Note that DNA-protein complexes also serve as immune stimuli to recruit and activate immune cells for bacterial clearance (*46*). For example, phagocytes can uptake NETs and may use different DNA-protein complexes to kill intracellular bacteria, but this situation is not explored in our current studies.

Despite its limited bactericidal potency, NE serves as an important serine protease to degrade extracellular matrix proteins and magnify proinflammatory responses against bacterial infection. Proteolytic activity of NE is regulated by serine protease inhibitors, with AAT being the predominant inhibitor in the lung lining fluids. Patients with AATD suffer from a hyperinflammatory state in the lung lining fluids, because of insufficient AAT levels (<2 μM) to neutralize the enzymatic activity of NE (*47*). Consequences of hyperactive NE include disruption of the lung epithelial barrier, decreased internalization of bacteria, and increased bacterial load (*48*). Pott *et al.* (*35*) found that AAT treatment can restrict the invasion of *P. aeruginosa* into respiratory epithelial cells. Later studies suggest that AAT may also inhibit the NE-mediated degradation of antimicrobial proteins, such as airway-secreted SPLUNC1 (*49*), immunoglobulins, and defensins (*50*). Histone, which is largely studied in an epigenetic context, is often overlooked in regards to their antimicrobial functions and even more so in their impact in AATD infections (*51*). NE has been shown to decrease the cytotoxicity of histone in NET clumps, but its impact on the antimicrobial potency of NETs has not been systematically investigated. Using DHCs as a simplified model of NETs, we confirmed that NE can degrade the most abundant NET protein (histone), reduce the bacteria-trapping ability of DHCs, and impair their antimicrobial activity. The presence of sufficient AAT can protect histones from being degraded by NE, leading to sustained bacterial trapping; reduction in internal CFUs, which suggested a reduced presence of bacteria inside epithelial cells; and enhanced overall antibacterial potency of DHNEs. These results indicate preservation of histones as one mechanism of how clinical supplementation of AAT reduces the bacterial load in patients with AATD.

Clinically, patients with AATD suffer from a complex disorder, arising from the imbalance between serine proteases and protease inhibitors, which also lead to broader immune dysfunction. Supplementation of AAT may contribute to improved host immunity against infection through protease-dependent and independent mechanisms (*52*), including but not limited to the suppressed apoptosis of neutrophils, alleviated damage to epithelial/endothelial cells, and coordinated release of cytokines and chemoattractant. Hence, in vivo studies on the effect of AAT on bacterial infection are always confounded by effects of AAT not associated with protease inhibition. Here, our NET-mimetic biomaterial model provides a defined minimal platform to show a causal link between the NE inhibitory activity of AAT and preservation of histone-mediated antimicrobial activity of NETs. Compared with animal studies, this analytical process excludes the complicated interference of NE-mediated activation/degradation of cytokines, chemoattractant, and non-NET proteins that occurs in vivo. While analysis of the bactericidal activity of DHCs with and without AAT are most directly relatable to AATD, the insights may also relate to other NET-abundant lung infections, such as COPD and CF. Further studies need to monitor the histone levels or the DNA/histone ratios in NET samples from infected lungs with hyperactive NE.

In summary, a material synthesis approach is introduced to dissect the antimicrobial activity of NETs. While we have previously reported on the synthesis of minimal NET-mimetic mesh-like materials composed of DNA and histones, this report further incorporates other crucial NET proteins such as NE and MPO as well as tissue environment factors that affect NET function, such as AAT, into the functional biomaterial modeling of NETs (*9*, *12*, *53*, *54*). Placing our focus on antibacterial activity against PAO1, we identified histone, a critical protein in epigenetics often overlooked in antibacterial research, as the most potent antimicrobial among the major NET proteins. We also show that NET-resident proteases, such as NE, degrade histones and decrease the positive charge of DHNEs, thereby impairing the bacterial trapping and killing function of our NET-mimetic material. We additionally studied the impact of incorporating AAT, a protease inhibitor of NE that also has direct immunomodulatory activity on cytokines and neutrophils. Physiologically relevant in vivo studies currently report conflicting results that lead to mechanistic ambiguity underlying AAT function. Thus, as a complementary approach, we developed a NET-mimetic system with controllable compositions of NE and AAT and clarified that AAT inhibition of NE-mediated histone degradation, per se, correlates with preservation of bactericidal activity. While this report focuses on the system’s antimicrobial activity, the concept and method of synthesizing NET-mimetic materials with defined compositions that can simulate NET functions in tissue environments open many new opportunities to gain insight on a range of NET-associated physiological effects and diseases.

## MATERIALS AND METHODS

### Culture medium

SCFM (*18*) was prepared by following published protocol. Briefly, 1.3 mM NaH_2_PO_4_, 1.25 mM Na_2_HPO_4_, 0.348 mM KNO_3_, 0.271 mM K_2_SO_4_, 2.281 mM NH_4_Cl, 14.943 mM KCl, 51.848 mM NaCl, 10 mM Mops, 1.446 mM serine, 1.549 mM glutamate, 1.661 mM proline, 1.203 mM glycine, 1.780 mM alanine, 1.117 mM valine, 0.633 mM methionine, 1.121 mM isoleucine, 1.609 mM leucine, 0.676 mM ornithine, 2.128 mM lysine, 0.306 mM arginine, 0.013 mM tryptophan, 0.827 mM aspartate, 0.802 mM tyrosine, 1.072 mM threonine, 0.16 mM cysteine, 0.53 mM phenylalanine, 0.519 mM histidine, 3 mM dextrose, 9.3 mM l-lactic acid, 1.754 mM CaCl_2_, 0.606 mM MgCl_2_, 0.0036 mM FeSO_4_, and 0.3 mM *N*-acetylglucosamine were dissolved in deionized water. The medium pH was adjusted to 7 using 2 M NaOH solution.

### Preparation of DHCs

Before preparing DHCs, concentrations of all proteins and DNA were measured with a microvolume ultraviolet-visible (UV-Vis) spectrophotometer (NanoDrop 2000, Thermo Fisher Scientific). Suspensions of DHCs were made with DNA (0.6 mg ml^−1^; salmon, Sigma-Aldrich, D1626) and one or more NET proteins at the following concentrations: 553 μg ml^−1^ of histone (calf thymus, Sigma-Aldrich, H9250), 111 μg ml^−1^ of LTF (human neutrophil, Athens, 16-14-120103), 80 μg ml^−1^ of NE (human neutrophil, Athens, 16-14-051200), 42.8 μg ml^−1^ of MPO (human neutrophil, Athens, 16-14-130000), 33.8 μg ml^−1^ of AZU (human neutrophil, Athens, 16-14-012621), and 35.3 μg ml^−1^ of CG (human neutrophil, Athens, 16-14-030107). Mixture were sonicated using a Q125 sonicator (Qsonica) for 5 s with sonication intensity set at 20% (*12*).

### Isolation of polymorphonuclear leukocytes and NETs

Peripheral blood was collected from healthy donators with their informed consent at Shanghai Sixth People’s Hospital Affiliated to Shanghai Jiao Tong University School of Medicine. The work was in accordance with and approved by the University Committee on biomedical research involving human participant at the Shanghai Jiao Tong University, Shanghai, China (E2022299I). Fresh peripheral blood from healthy donors was collected with EDTA as an anticoagulant. A piece of 5-ml blood sample was carefully layered over 5 ml of Polymorphprep in 15-ml tubes. The sample was then centrifuged at 550*g* for 30 min, yielding a polymorphonuclear leukocyte (PMN)–rich cell band. The PMNs were transferred into RPMI 1640 supplemented with 10% fetal bovine serum to restore normal osmolality. Remnant red blood cells were lysed using a cell lysis buffer (Sigma-Aldrich), followed by centrifugation at 500*g* for 10 min. Purified PMNs were resuspended in serum-free RPMI 1640 and kept on a flat tissue culture dish in an incubator.

Living neutrophils were stimulated with 500 nM phorbol 12-myristate 13-acetate (PMA) and cultured in a cell incubator for 4 to 5 hours to allow for NETosis. After the allotted time, the NET-rich and cell-rich layers were collected by washing the plate with cold phosphate-buffered saline (PBS). A cell-free NET-rich supernatant could be obtained by centrifuging the cold mixture at 500*g* for 10 min at 4°C. Further centrifugation of the supernatant at 18,000*g* for 10 min at 4°C forms pellets of NETs. The purified NETs were washed again with cold PBS, and the NET DNA concentration was measured using NanoDrop 2000 (*55*).

### Bacterial culture and antimicrobial activity test

#### Bacteria

The plasmid pUCP20 containing the reporter gene encoding mCherry was electroporated into *P. aeruginosa* strain PAO1 for 1.8 kV, 2.8 ms at 4°C. The resulting transformants were streaked on a Mueller-Hinton agar plate supplemented with ampicillin (200 μg ml^−1^). After overnight culture at 37°C, the mCherry-PAO1 transformants expressing the red fluorescent protein (Ex/Em = 590/630 nm) were resuspended in SCFM supplemented with 20% glycerol and stored at −80°C.

#### Culture method

Frozen bacterial glycerol stock containing mCherry-PAO1 was streaked on a tryptic soy agar (TSA) plate. After overnight incubation, one to two bacterial colonies were scratched from agar plates and inoculated into 1 ml of SCFM. The PAO1 bacteria were incubated inside a bacterial shaker (200 rpm) at 37°C for about 4 hours, until the OD_600_ of the bacterial culture reached ~0.3 to 0.6. Subsequently, the bacterial culture was diluted with SCFM until OD_600_ = 0.01.

#### Bacterial trapping assay

A total of 100 μl of suspension of DHNEs was allocated into a 96-well microplate. The DHNE clumps were spun down at 2000*g* for 15 min (4°C). PAO1 was seeded at a density of 10^8^ CFU ml^−1^ and was also spun down at 2000*g* for another 15 min. Next, PAO1 was incubated with DHNE clumps at 37°C for 30 min, which enables the untrapped PAO1 to detach from the DHNEs. Last, the supernatant containing planktonic untrapped PAO1 was aspirated, and the bacteria trapped on the NETs were quantified from the mCherry fluorescence. As a blank control, the RFUs of all bacteria in the culture were measured by spinning down bacteria onto the poly-lysine–coated microplates. The trapping efficiency was calculated as the RFU ratio of bacteria trapped on the DHCs relative to all bacteria.

#### Bacterial CFUs

A total of 10^5^ PAO1 cells was seeded with a single NET-derived component in 100 μl of PBS. After coculture for 2 hours at 37°C, the bacteria culture was diluted 1000-fold with PBS, transferred onto TSA plates, and incubated overnight. The number of CFU counts in the presence of different NET-derived components were numerated.

#### Bacterial growth curve

The diluted bacterial culture (100 μl) was mixed with an equal volume of DHNE suspension (supplemented with or without NE inhibitor) before being transferred into a 96-well microplate. Bacterial growth curves of PAO1 in SCFM were obtained by measuring RFUs with a microplate reader (Biotek Synergy HT; Ex/Em = 590/630 nm, 37°C) for 24 hours.

### Cell culture and antimicrobial activity test

#### Cell

The human lung alveolar epithelial cell line A549 was obtained from the Procell Life Science & Technology Company. A549 was cultured in medium composed of Ham’s F12 medium (Corning, USA) supplemented with 10% fetal bovine serum (Gibco, USA) and 1% penicillin-streptomycin (Gibco, USA). Incubations were conducted in an incubator at 37°C supplied with 5% CO_2_.

#### Antimicrobial activity test

A monolayer culture of A549 cells was done in 24-well microplates. DHNEs with and without AAT were prepared and aged for 1 hour. A549 cell culture medium was discarded and replaced by a mixture of 10^4^ per well of PAO1 and aged DHNEs. A549 and PAO1 were cocultured and supplemented with DHNEs (±AAT) for 1.5 hours, and the CFUs of survived PAO1 after coculturing were numerated.

#### Gentamicin protection assay

This assay is to determine the number of intracellular bacteria in A549 cells. A549 cells (200,000 per well) were first cultured into 12-well microplates to form a monolayer. PAO1 (10^5^ CFU per well), DHNEs (DHC: 0.1 mg ml^−1^, NE: 0.15 μM), and DHNEs with AAT (1 μM) were subsequently seeded into the cell culture plate and incubated for 30 min. Supernatants were discarded, and the cell layer was washed with PBS for three times. Gentamicin (0.2 mg ml^−1^) was added to the culture medium and incubated for 1.5 hours to kill extracellular bacteria. The cell culture plates were washed by PBS twice to remove gentamicin. Triton X-100 (1%) was added to disrupt the cell membrane to release the intracellular PAO1. Last, CFUs of released PAO1 from A549 was numerated.

### ELISA and enzyme activity test

DHNEs and DHMPOs were prepared using the method specified in the “Preparation of DHCs” section. After incorporation of NE (or MPO), suspensions of the DHCs were spun at 5000*g* for 10 min. Supernatants were collected for enzyme activity assays using commercialized kits (NE: Abcam, MPO: Cayman). The background signal was subtracted using NE-free (or MPO-free) DHC samples as blank controls.

To determine the amount of NE incorporated in the DHNEs, the supernatant and the DHNE clumps were separately collected. The DHNE clumps were dissolved with DNase I (1 mg ml^−1^). After NE was released, its concentration was quantified using a human NE ELISA kit (Fankew, F0640-B).

### Zeta potential measurement

NE and its inhibitor were mixed together before adding desired concentrations into DHC suspensions. The mixture was diluted in Hank’s balanced salt solution at a ratio of 1:20 [DNA (20 μg ml^−1^)] and injected into disposable folded capillary cells (Malvern). The dynamic change in the zeta potential of the DHNEs (with and without inhibitor) was monitored using a Zetasizer (Malvern) at 37°C.

### Histone degradation test

To visualize the degradation of histone in DHNEs, the calf thymus histone (Sigma-Aldrich, H9250) was labeled with FITC (Sigma-Aldrich) and dialyzed against SCFM using a Float-α-Lyzer G2 device (weight-average molecular weight cutoff of 500 to 1000 Da; Sigma-Aldrich). Purified FITC-histone was mixed with DNA to form DHCs. Five microliters of DHNE suspension was spotted into a 96-well microplate and dried in a desiccator overnight. The resulting pellet was rehydrated with SCFM supplemented with (or without) NE and AAT at 37°C for 10 min. After washing the DHNEs with SCFM, the remaining structure was observed using an inverted fluorescence microscope (DMi8, Leica).

Quantitative degradation tests on DHNEs were conducted by measuring the OD_600_ of the DHNE suspension (with or without the NE inhibitor AAT) as a function of degradation time. DHCs were formed in which the concentration of DNA is maintained at 0.6 mg ml^−1^, but the histone concentration is varied from 0 to 0.5 mg ml^−1^. The DHCs were homogenized using an ultrasonicator for 5 s (sonication intensity set at 20%) before undergoing OD_600_ measurements. To generate a calibration curve, different concentrations of DHCs were prepared, and their optical density was independently measured using a UV-Vis spectrophotometer (Nanodrop OneC). To measure the dynamic degradation of histone in DHCs, DHC samples (with and without AAT) were incubated at 37°C for different periods of time (up to 6 hours), and their optical densities were monitored. Immediately before taking each OD_600_ measurement, the DHNE mixture was carefully homogenized to avoid sedimentation of the DHNE clumps. The concentration of the nondegraded histone was deduced from the reduction in turbidity of the colloidal suspension.

In Western blot, purified histone (0.6 mg ml^−1^) or DNA-proteins complexes [DHNEs contain DNA (0.6 mg ml^−1^), histone (0.553 mg ml^−1^), 1 μM NE, and 6 μM AAT] were prepared by heating at 100°C for 5 min after mixing with loading buffer. Ten microliters of each samples was loaded onto 15% SDS–polyacrylamide gel electrophoresis and underwent electrophoresis. Proteins were then transferred to 0.45 μm of PVDF membranes (Millipore, USA). The unspecific antibody binding sites were blocked by incubating the membrane at 5% skim milk. Then, the membrane was incubated with histone H3 monoclonal antibodies (1:1500 dilution ratio; Beyotime, China) overnight at 4°C. A horseradish peroxidase–labeled secondary antibody (1:5000 dilution rate; Cell Signaling Technology, USA) was added by incubating at 20°C for 2 hours. Protein bands were detected with an enhanced chemiluminescence kit (Beyotime, China) and photographed with a visualization system (Tannon, China).

### Scanning electron microscopy

Suspensions of DHNEs (with or without AAT) were cocultured with PAO1 on silicon wafers for 2 hours before 4% paraformaldehyde fixation for 1 hour at room temperature and a subsequent PBS wash. The fixed samples were then dehydrated in a series of ethanol-water mixtures with increasing ethanol volumetric fraction (25, 50, 75, 90, 95, and 100%). Afterward, the samples were transferred into hexamethyldisilane and vacuumed overnight. Before SEM imaging, the samples were sputter-coated with gold (~5 nm in thickness) using a Hummer 6 gold/palladium sputterer. SEM images were taken using Hitachi SU8230 with an acceleration voltage of 0.5 to 1 kV (the electron beam current is set as 10 nA).

### Statistical analysis

All the results were described as means ± SD. The data were collected and analyzed with one-way analysis of variance (ANOVA). Statistical significance is denoted by ns (not significant), *P < 0.05, **P < 0.01, ***P < 0.001, and ****P < 0.0001.

## Notes

View/request a protocol for this paper from *Bio-protocol*.
