# Supplementary material for: Synthetic neutrophil extracellular traps dissect bactericidal contribution of NETs under regulation of α-1-antitrypsin
Source: Sci Adv. 2023 Apr 28;9(17):eadf2445. doi: 10.1126/sciadv.adf2445 (PMC10146876; doi:10.1126/sciadv.adf2445)
Supplement: Supplementary file 1 — Supplementary Text Figs. S1 to S9 [file sciadv.adf2445_sm.pdf]

Science Advances
AAAS

Supplementary Materials for

Synthetic neutrophil extracellular traps dissect bactericidal contribution of NETs under regulation of α-1-antitrypsin

Ting Yang *et al.*

Corresponding author: Yang Song, nanosurface@sjtu.edu.cn; Shuichi Takayama, takayama@gatech.edu

Sci. Adv. **9**, eadf2445 (2023)
DOI: 10.1126/sciadv.adf2445

This PDF file includes:

Supplementary Text
Figs. S1 to S9

Supplementary Text

Staining Methods

NETs (about 600 μg) were isolated from PMA stimulated neutrophils from 25 mL human blood using previously published protocol, resuspended in 1.5 mL PBS. The NETs (containing neutrophil debris) were collected, resuspended and spin down (2200 g, 15 mins) to form a film in chambered coverslip (μ-Slide 8 Well, ibidi). The NET film (a) without NE treatment, (b) incubated with 1 μM NE for 30 mins at 37℃, or (c) incubated with 1μM NE and 5μM NE inhibitor AAT for 30 mins at 37 ℃. All samples were fixed with 4% PFA, washed by PBS before immunofluorescence straining. These NET samples (containing 100 μg mL^{-1} NET DNA) were incubated with 4wt% bovine serum albumin (BSA) for 30mins at 37 ℃, followed by incubation with 1wt% BSA + 5μg mL^{-1} primary histone antibody (Mouse anti-histone H2B antibody, Abcam 52484) + 5μg mL^{-1} NE antibody (Rabbit antibody to neutrophil elastase, Abcam 68672) at 4 ℃ overnight, and repeated washing by 1wt% BSA for 15mins. Subsequently, the samples were incubated with 1wt% BSA+ 5μg mL^{-1} FITC- goat anti-mouse lgG1 secondary antibody (NOVUS, NB7510) + 10μg mL^{-1} goat anti-rabbit lgG tagged with texas red (NB120-6719) at room temperature for 2 hours. DNA in neutrophil-NETs was labelled with 1ug mL^{-1} DAPI, washed with PBS before LSCM imaging.

Tissue Cell Culture and Cytotoxicity

Human umbilical vein endothelial cells (HUVECs) were cultured in RPMI 1640 or 1640 supplemented with 10% FBS (Gibco) and 1% penicillin-streptomycin. To test cytotoxicity of NET-derived components, HUVECs were seeded into 96-well microplates at a density of 10,000 cells per well and incubated at 37 ℃ in 5% CO_2 overnight. At this stage, the cells were treated with NET-derived components, including NE, histone, DHCs and DHNEs. AAT was added to reflect the effects of NE inhibitor on the cytotoxicity of DHNEs. After 24 hours, cells were washed with PBS three times before incubation with 10% CCK-8 in RPMI 1640 for an additional 2 hours. The optical absorbance was measured at 450 nm wavelength using a microplate reader. Each test condition had six replicates for this CCK-8 cell viability assay.

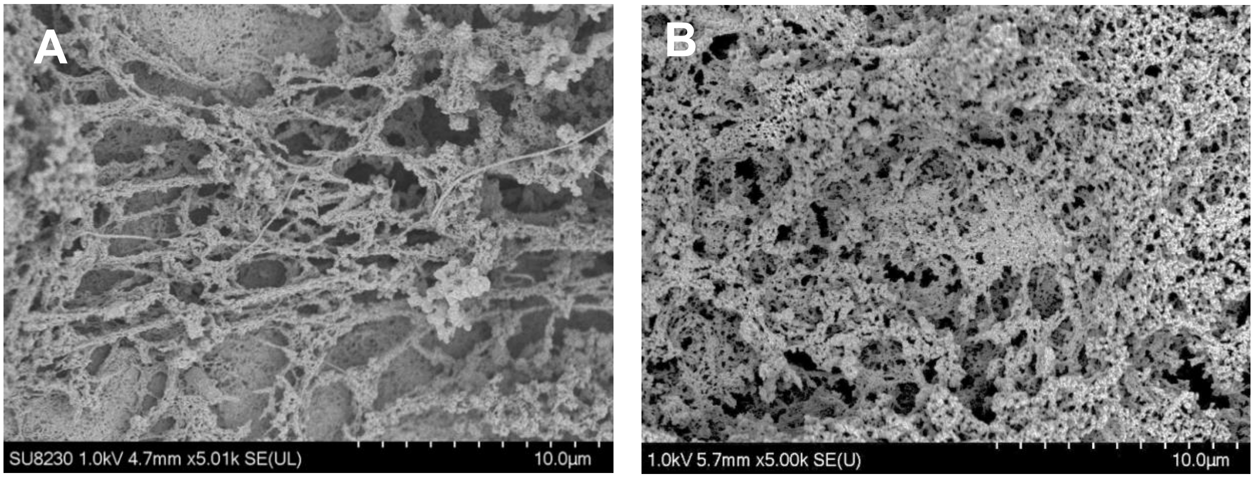

Fig. S1.

SEM images of synthetic DHC with NET-like structure. (a) SEM image of low concentration DHC (0.1 mg mL $^{-1}$). (b) SEM images of high concentration DHC (0.6 mg mL $^{-1}$). Low concentration of DHC had loose fibers and the fiber diameter was approximately 50 nm. At high concentration, the fiber network is denser and the fiber diameter can be as high as 200 nm.

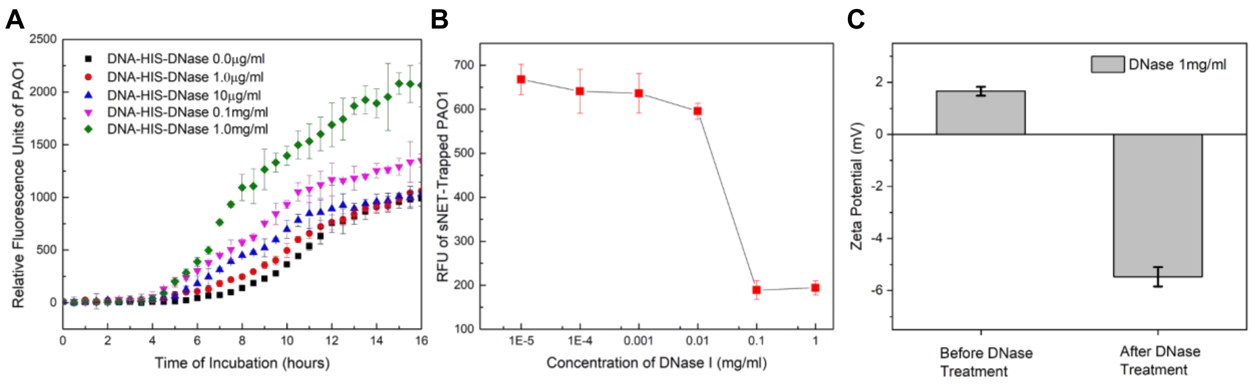

Fig. S2.

DNase effect on DHC inhibition and trapping of PAO1. (a) Growth curves of PAO1 in presence of DHCs with DNase concentration ranging from 0 to 1 mg mL^{-1}. (b) Effect of DNase on bacterial trapping ability of DHCs. (c) Zeta potential of DHCs before and after 1 mg mL^{-1} DNase treatment.

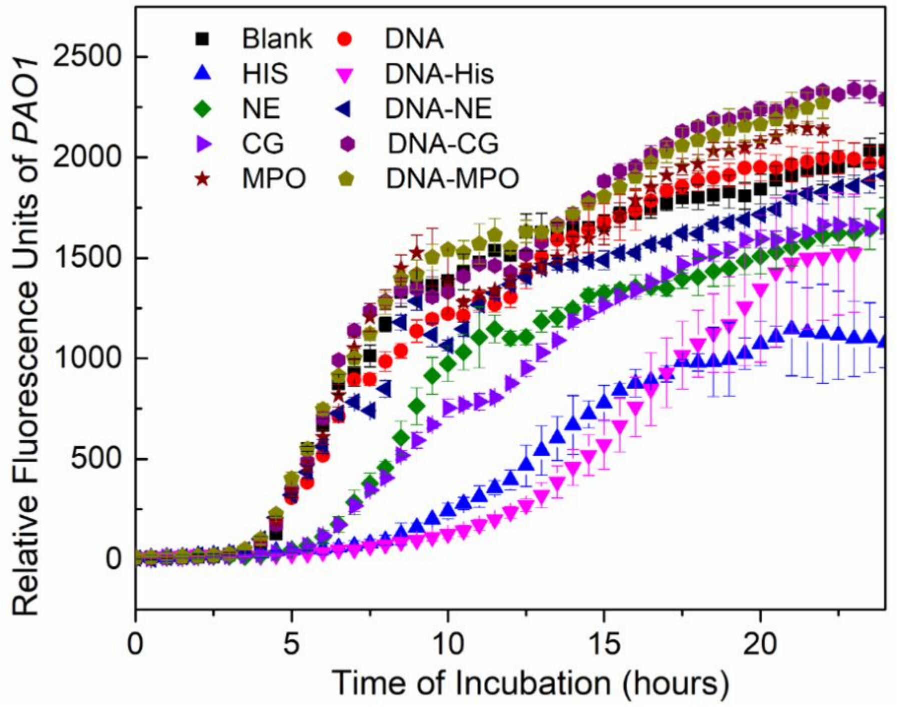

Fig. S3.

Growth curves of PAO1 in SCFM supplemented with different NET proteins and DNA-protein complexes. Concentrations of DNA and NET proteins used in this study are listed in Table 1.

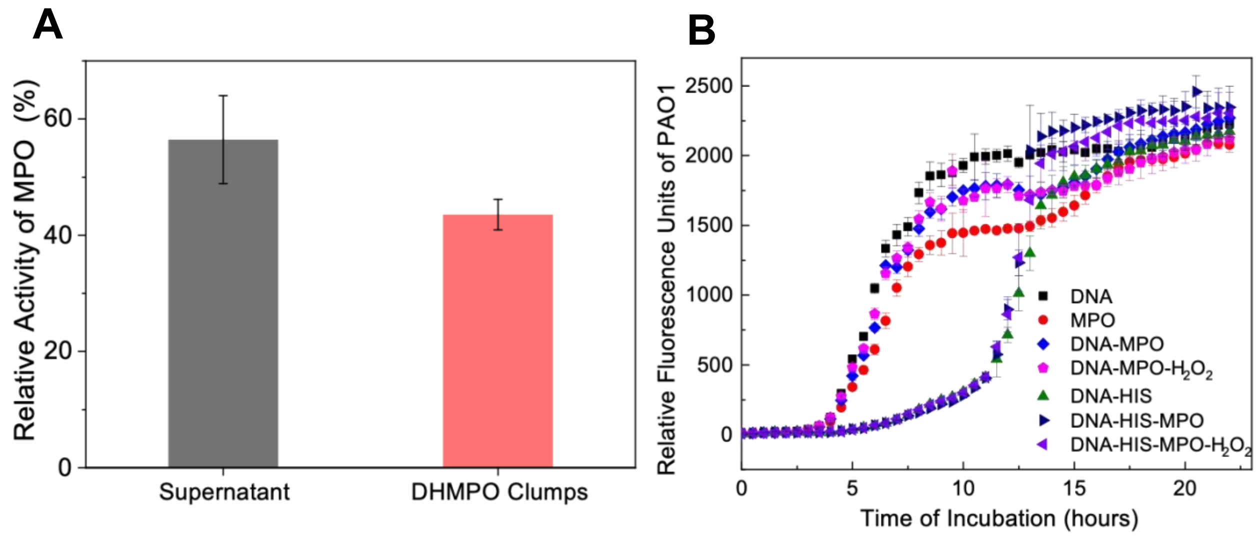

Fig. S4.

MPO inclusion in DHCs. (a) The relative amount of MPO incorporated in the DHC. (b) Growth curves of PAO1 in SCFM in the presence of DHCs with MPO and H_2O_2. MPO-incorporated DHCs do not further change the growth rate of PAO1 relative to MPO-free DHCs. Adding H_2O_2 to facilitate bleach production by MPO, at physiological concentrations of CF disease, also does not change the growth rate of PAO1. Extracellular concentration of H_2O_2 in CF airway fluids varies from 0 μM up to 5 μM.

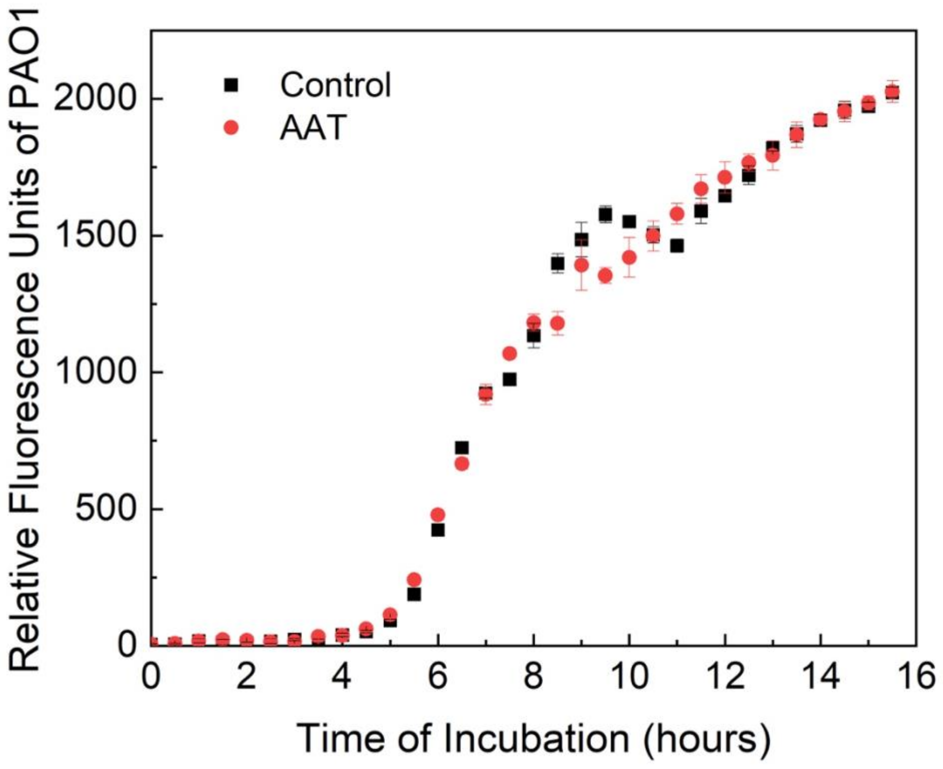

Fig. S5.

Growth curve of PAO1 cocultured with AAT. Bacterial growth curves were generated by culturing seeded PAO1 (10^7 CFU mL^{-1}) with or without AAT (5 μM) for 16 hours at 37 ˚C.

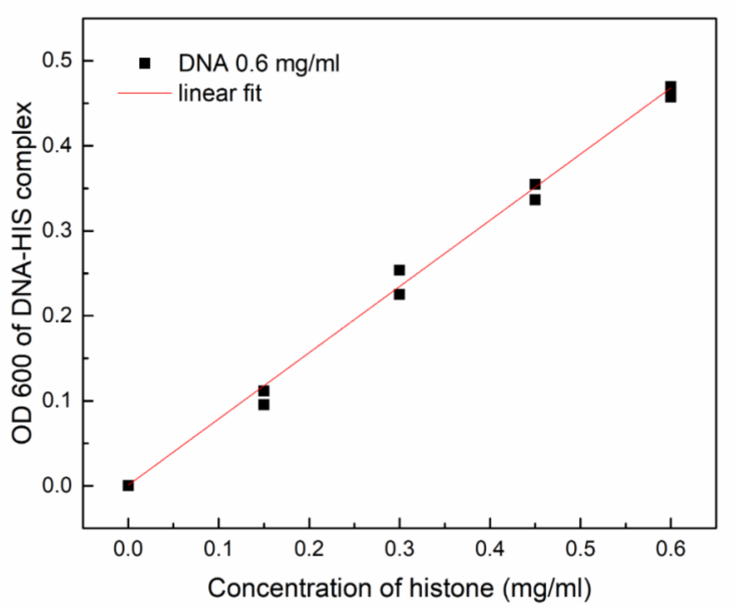

Fig. S6.

Optical density (OD_{600}) of homogenized DHC linearly decreases with histone concentration.

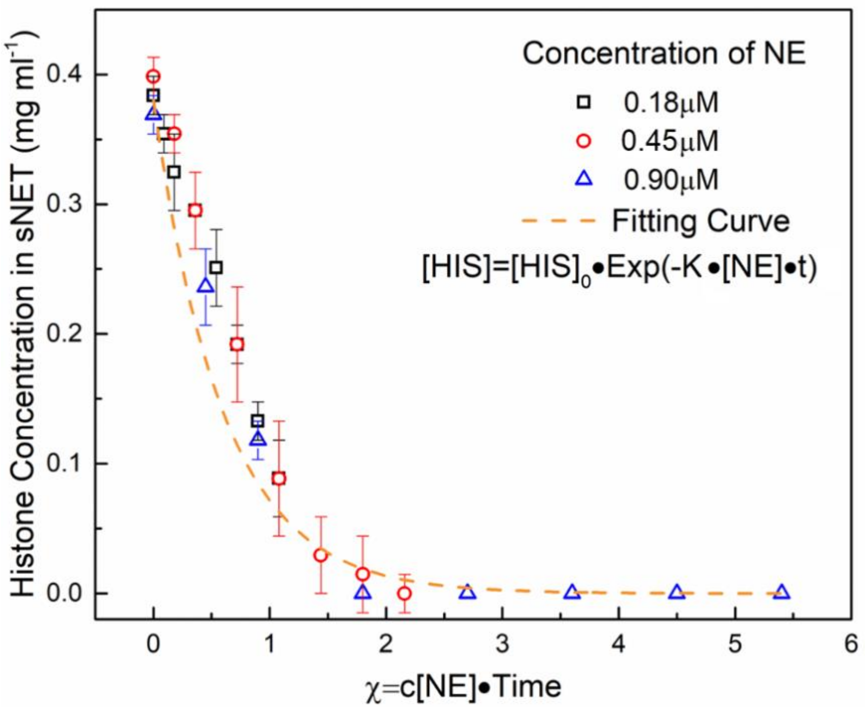

Fig. S7.

Experimental determination of K_{NE} in CF sputum mimetic media. (a) K_{NE} is determined by measuring the degradation kinetics of histone in SCFM and fit the plots according to Equation 1. (b) Effect of viscosity of the SCFM on the K_{NE}. Viscosity of the SCFM is adjusted by adding glycerol from 0vol% to 80vol%. Dynamic viscosity of CF sputum is on the order of 10-100 mPa S, and the estimated K_{NE} is on the order of 0.1.

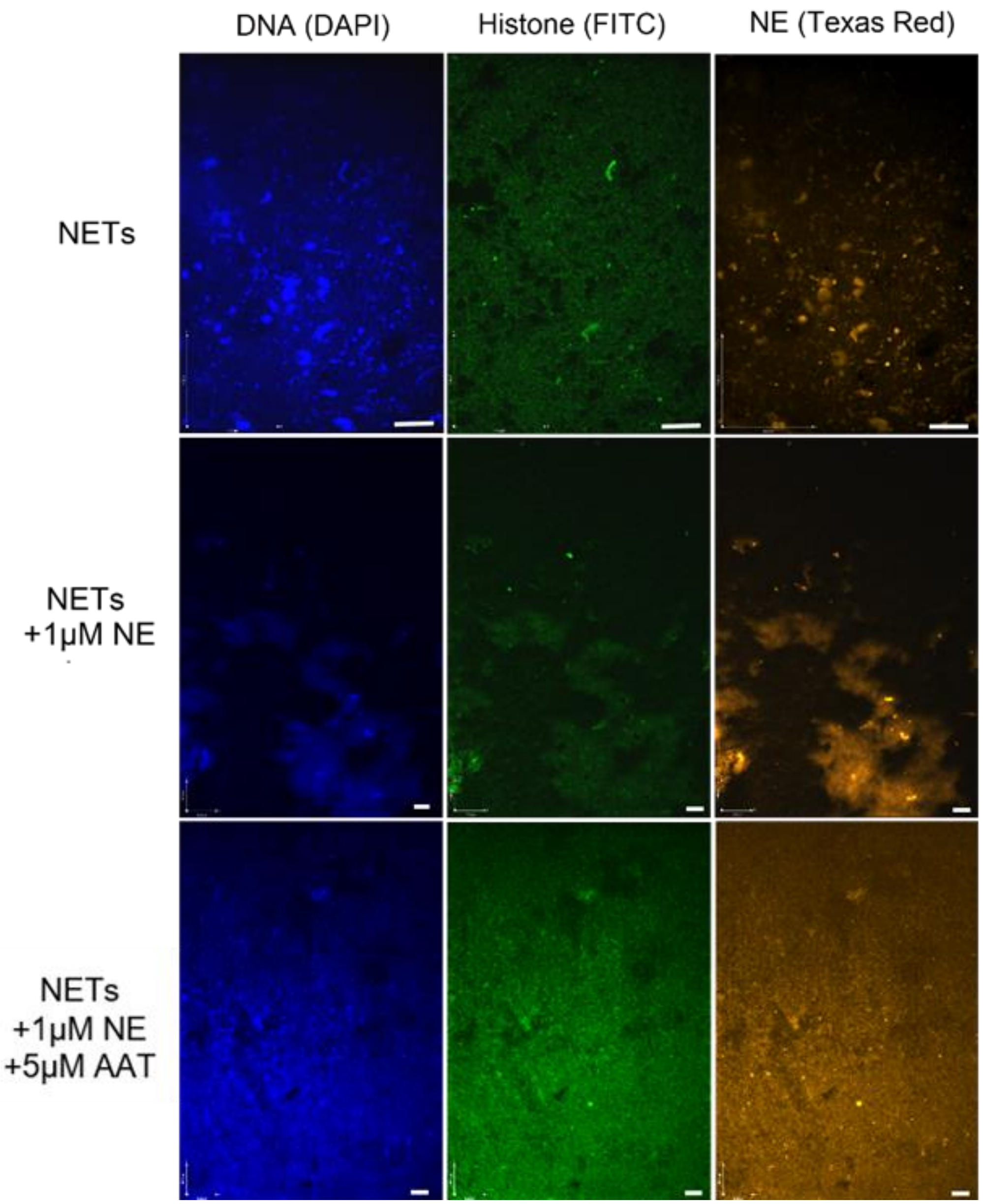

Fig. S8.

Laser scanning confocal microscopy (LSCM) images showing hyperactivity of NE induces degradation of histone in endogenous NETs. (top row) NE and histone were colocalized with DNA in the NET structures. (middle) After addition of 1 µM NE (as in CF airway fluids), the NET structure is partially dissolved after incubation at 37 ˚C for 30 min. After washing and immunofluorescence staining, much less histone (green) was detected in the NET structure, suggesting NE-degraded histone. (bottom) When 1 µM NE and 5 µM AAT were added simultaneously to NETs before incubation, the NE activity was completely neutralized by AAT. Consequently, the histone was preserved in the NET structure. Scale bars, 20 µm.

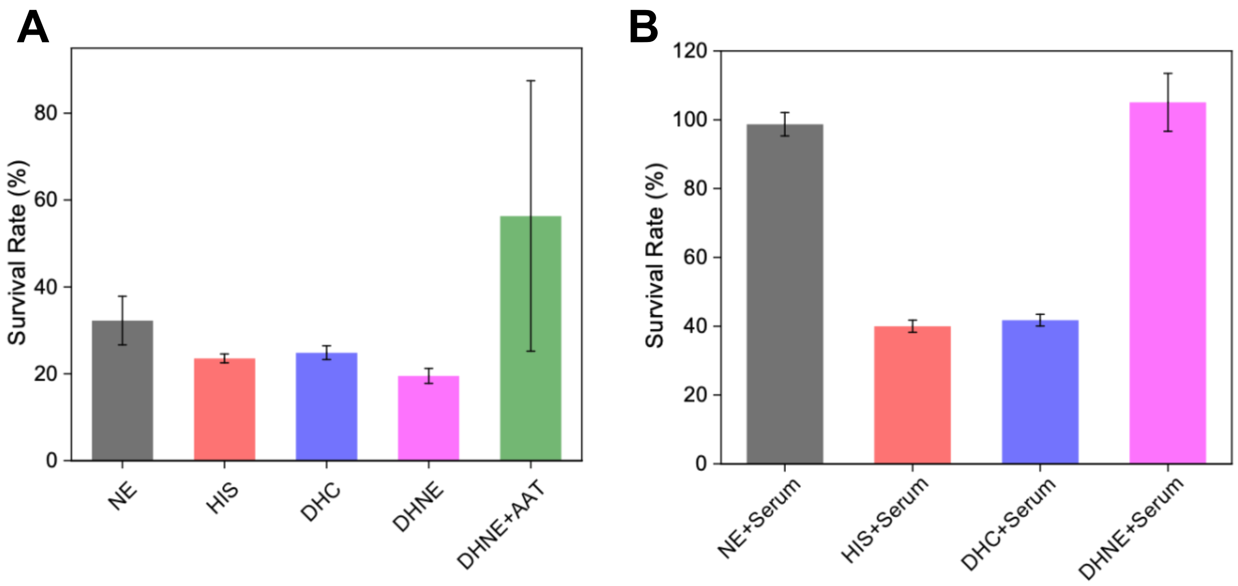

Fig. S9.

Cytotoxicity of NETs is regulated by serine protease inhibitor from tissue environment. (a) Viability of human umbilical vein endothelial cells (HUVECs) in serum-free culture medium supplemented with different combinations of NET components (see Table 1 in main text for concentrations of each component). (b) Viability of HUVECs in serum-containing cell culture medium supplemented with different combinations of NET components.

Excessive NE production, either from NETs or from neutrophil degranulation, cause chronic damage to lung epithelial cells. Using a high level of synthetic DHNEs as a disease model for aberrant NET clumps found in CF, we find that DHNEs cause substantial death of human umbilical vein endothelial cells (HUVECs) in serum-free culture medium, but not in serum-supplemented medium. This is consistent with the fact that blood contains a higher level of elastase inhibitor than the lung lining fluids. In a chronic inflammation environment such as the later-stage CF lung, the activity of NE is increased up to 2.3 μM, and our model suggests this would lead to rapid degradation of bacterial histone, severely impairing the bactericidal activity of NETs. The evidence and analysis here indicate that NE and its inhibitor coordinately regulate the homeostatic level of histone in healthy lungs, but hyperactive NE could break down this feedback regulation regime.
